# Present State of Medicine in Italy, Principally from the Writings of Tommasini

**Published:** 1822-03-01

**Authors:** 


### PRESENT STATE OF MEDICINE IN ITALY.

1. *Della Nuova Dottrina Medica Italiana, &c.* Del Professore G. Tommasini, Firenze, 1817, pp. 98.

2. *Dell' Infiammizione e della Febbre Continua-considerazioni Patologico-Pratiche.* Di G. Tommasini. Pisa 1820, pp, 272.

3. *Prospetto De' Resultamenti ottenuti nella Clinica Medica della Pontificiu Universita' di Bologna nel Corso di un Triennio Scholastico. Discorso premesso alle Lezioni Medico-pratiche dell' anno Scholastico* 1819, 1820. Dal Professore Giacomo Tommasini. Pisa 1820, pp. 48.

Although the avowed and actual object of this Journal is, to present to its readers an analytical record of practical medicine, as exhibited in the never-failing produce of the press;—neither we, nor our patrons, are so strait-laced as to sacrifice all other considerations of curiosity or interest to that of mere practical utility. When wearied, therefore, with the everlasting labours of our diurnal calling,—in copying in miniature on our pages, the full-length pictures, that meet our eyes, on all sides, from the pulse-feeling fingers of practical men,—we take no shame to ourselves, nor think it requisite to crave the pardon of our readers, if we occasionally, indulge ourselves, and them, with sketches of a less sombre and a lighter character. Of this class is the notice which we take, from time to time, of those theories and speculations, which, God knows, will never be of any other practical benefit, but that of keeping the blue devils from their authors, during the period of their concoction; and "which play round the head, but come not to the heart" of such musty subjects as ourselves, whose taste and talent for theory have long been spoilt by the ill-mannered and uncourtly obstinacy of vulgar disease. Of a kindred, but less futile, class of subjects are those, which relate to the state of medical opinion and practice in different countries; the progress of medical literature, and the biography of illustrious men. To the former of these subjects, the present sketch will be devoted; and we leave the interest, if not the utility, of the details to plead for themselves.

Italy—the very name of Italy—must possess an interest and a charm to all that look upon that lovely and famous land, whatever be the object of their contemplation or inquiry. Our early intimacy with the nations of classical antiquity, through the proxy of their immortal authors, gives them peculiar claims to our attention; and, from that identification of feeling with the objects of regard, which can only arise in the generous breast of youth, we can never look upon them afterwards, even in their evil days and most degraded fortunes, but with a tenderness partaking of the love for a native land. It cannot, therefore, be supposed, that our fraternity, whose official language is still that of ancient Rome, can look with indifference on the existing condition of their profession in Italy. And it must be delightful to them, and to every friend to humanity, to know, that the present state of medical science in that country is, comparatively with former periods, most flourishing. In fact, it is such, as to bear comparison with that of any country in Europe; and is certainly very superior, both in principle and practice, to that of the great majority of continental states. This comparative excellence, however, is, as we shall shortly see, of no very antient date; and the whole science may be truly said to possess, at this very moment, the boisterous vigour and noisy zeal of youth.

Italy has always been the prey of conquerors; yet none of her masters ever established a more complete authority over her political fortunes, than one of our own countrymen extended, sometime ago, over her medical independence. John Brown, (like his gifted countryman Robert Burns, at once the glory and disgrace of Scotland,) was destined to receive from foreign nations the unbounded honours, to which both himself and his friends considered his genius as entitled; but which were most pertinaciously withheld from him in his native country. While he was struggling for existence in Edinburgh, against the cabals and persecutions of his opponents, and the more fatal array of his own violent and illregulated passions;—or was subsisting in London on the charity of his few remaining friends,—with no patients, and scarcely a disciple;—his *New Medical Doctrine* was spreading like wildfire over the Continent; and in Italy, more especially, was subduing to its influence, alike the truths and errors of former systems, and fixing itself, like a delightful and unresisted spell, on the minds of the younger members of the profession. In a very few years after the death of its author, while the slight impression which the New Doctrine had made in England was (with the exception of some of its principles, which can never die,) almost entirely effaced, in Italy the whole of it was established as the catholick rule both of reasoning and practice.

It is needless to observe that the author of such a revolution must have been a great man; and it is equally needless to deny that, *as a system*, that of Dr. Brown equals, if not exceeds, in originality and ingenuity, any that have been promulgated in medicine. No one will, assuredly, accuse us of being Brunonians; nor will those acquainted with our critical labours, believe us capable of underrating the merits of Dr. Cullen; yet we conceive it will be now universally admitted, that the merits of the latter, as a theorist, are insignificant when compared with those of his great but unfortunate rival. But for the extreme value of his admirable practical writings, the name of Dr. Cullen would only now be ranked in the class of minor speculators in medicine. Brown, on the contrary,—LIO SCOZZESE—THE GREAT SCOTSMAN as he is called in Italy,—like the sculptor of antiquity, who engraved his own emblem on a part of one of his immortal works,—has so deeply impressed the stamp of his genius on medicine, that it can never be effaced, but by the destruction of the science itself. And yet, however humiliating the confession, we must admit, that his doctrine has been the source and sanction of a practice most injurious to mankind. The offspring of a mind more versed in abstract science than in practical medicine, its beautiful simplicity was, in few cases, adapted to the explanation of the complicated phenomena of disease; and, even when just, its principles, when rigidly followed in practice, often led to the most fatal consequences. As necessary inferences from the doctrine of excitability, it followed that almost all diseases either were originally, or speedily became *asthenic*, or of *debility*, requiring stimulants for their cure; a class, which was still farther enlarged by that part of the doctrine, which considered general diseases as of the same nature as their causes,—and local affections as necessarily of the same nature as the existing diathesis,—*sthenic*, or *asthenic*. Accordingly, in Italy, during the dominion of Brownism, all fevers, all acute inflammatory affections in their latter stages, and all chronic inflammations, were considered as diseases of debility, and treated with stimulants. The consequences were such as we can now very easily conceive. And it is, perhaps, on the whole, fortunate, that the doctrine and practice of stimulation were carried to the extreme they were; as the glaring and disastrous results eventually sufficed to open the eyes of the profession to the true state of things before them, and induced those to listen to the pleadings of nature, whose ears had been too long closed by the enchantments of a false philosophy.

Signal and complete as was the triumph of the Brunonian system in Italy, still, it is not to be supposed that it was effected without opposition; nor that there were wanting here, as in other conquests, some stubborn spirits, who still clung to antient opinions, and yielded a willing homage to the names and objects of former veneration. It was not, however, before the year 1800, that any thing like a systematic opposition was made to the established doctrines. And this appears to have been first done by *Rassori,* in his work on the epidemic petechial fever of Genoa. This was followed up by many publications of various physicians, in different parts of Italy; all of which are chronologically arranged in Tomassini's pamphlet. This author, himself, has been for many years, one of the most successful and distinguished opponents of the Brunonian system, and has, by his various writings, contributed largely to the establishment of the doctrine and practice which have now, for several years, very generally superseded it.

This New Doctrine, as it is called,—for the Brunonian, which in the foregoing observations we have been denominating *new*, is now named *the Old*,—is held up by the Italian writers as something original; and its discovery claimed for Italy, as if conferring on it the greatest honour. We shall presently see, however, that its principles are a mere modification of the systems of Brown and other theorists, and its practice in a great measure such as has been established for many years in this country. This, indeed, is distinctly avowed as the child of Brownism, but of a regenerate branch, and purged from all the frailties and vices of its sire.

The following is the pompous style in which it is announced in our author's pamphlet.

"The New Italian Medical Doctrine has created an epoch, and will undoubtedly hold a distinguished station in the history of medicine. The offspring of Solidism and Brownism, it is more simple than the doctrines of Hoffman, Baglivi, or Cullen, inasmuch as it abjures the many fruitless suppositions and questions which so long perplexed the followers of these great men, involving them in the obscure research of causes, and misleading them from the more tranquil study of effects, and from that plain method of induction which can alone be the groundwork of the medical art. The new doctrine is better calculated than the Brunonian to inspire confidence at the bed-side of sickness, because it does not reject many of the old and established modes of treatment, because it proscribes all abstract opinions, and because, having grown up more in practice than in study, it rejects many of the palpable errors which the pride of theory maintained against the commonest observation of facts." *Della Nuov. Dott. p.* 3.

"This New Doctrine" he says in another place, "was born in Italy; on this soil, ever fruitful in useful discoveries in every branch of science and art, it has grown up; and it looks to the united zeal of Italian physicians, for its highest possible perfection." *Ib. p.* 43.

In Italy, the very great contrast between the new doctrines and the old,—and yet more, between the new practice and the old,—naturally give to the former a great appearance of novelty; but in this country, in which the Brunonian system was never prevalent, these pretensions cannot but seem overstrained.

This will appear sufficiently obvious, from the following brief sketch of the New Doctrine, which we shall give without noticing, in general, its relation to former and existing systems.

Agents are conceived to operate on the living system in two different and opposite ways; the one set (the *stimuli* of Dr. Brown) exhausting the excitability, and thus producing excitement; the other (the *sedatives* of the Cullenian school) lessening the excitement without any previous or accompanying stimulant operation whatever, and in a manner equally direct and independent. The former class of agents are called *stimulants,* the latter, *contrastimulants;* and the general state of the system, or *diathesis*, produced by their respective operation, is called *stimulant* and *contrastimulant*.

The immense Brunonian class of diseases from indirect debility, is reduced in the new system, to a very insignificant number indeed; and the great influence of this, as a general source of diseases, is declared an illusion. Thus almost all fevers, and all inflammatory affections, in every stage, are considered, by the new school, as uniformly and invariably sthenic. In the New Doctrine, the nature of general diseases is considered (in opposition to the scheme of Brown) to have no necessary dependence on the nature of the cause; and local affections no necessary similarity to the existing diathesis. Thus, a sedative power may give rise to a disease of excitement, and an acute local inflammation may arise in a most debilitated system.

The class of agents named contrastimulants are thus described by Tommasini:—

"They act on the living fibre in a manner directly the reverse of stimuli, and immediately produce those effects on the excitability which Brown derives negatively from the diminution of stimulation. They remove the effects of excessive stimulation, and without any (necessary) evacuation; and, when used in excess, produce diseases which can only be removed by the use of stimuli: in their remedial effects, therefore, they operate like blood-letting and purgatives, and other evacuating measures."

The two classes of agents, stimulants, and contrastimulants, reciprocally correct each other's effects. The measure of the existing diathesis is afforded by its capacity to bear agents of the opposite class; and this degree of tolerance is a much better index of the nature and degree of the diathesis than the symptoms are.

A condition of the system neither stimulant nor contrastimulant may exist, consisting rather of a disturbance of action, than of an increase or diminution of action. This state is called *irritation*, and the diathesis *irritative*. The number of diseases depending upon this state are very few; the great majority belonging to the stimulant diathesis.

Pain, and certain other nervous affections, exert a great influence in modifying diseases. In acute diseases, these sensorial affections often act as contrastimulants, forbidding, for a time, the employment of other contrastimulants, which are indicated both before their occurrence and after they have passed off.

In the new doctrine, the phenomena of inflammation hold a most conspicuous place; and as well on this account, as because the subject can never be too much impressed on the minds of practitioners, we shall notice this part of the system somewhat more at length. With this view we shall avail ourselves of the larger work on inflammation, by Tommasini: and, in glancing over it a second time, shall put down a few of the more prominent subjects discussed, in the order of their occurrence, and without much connexion.

All inflammation is the simple product of excess of stimulus; all its characters are traceable to this, from the "primissimo rubore" to the extreme of disorganization. All inflammations, therefore, are uniformly, and in all circumstances, sthenic; and as the phenomena of the greater number of fevers, and of all the phlegmasiæ, are (contrary to the dogma of Brown) considered to depend on a primary local inflammation, this doctrine must be of immense importance in the practice of medicine. Perhaps no part, once inflamed, ever returns to its original soundness, although it appears to do so to our imperfect senses. This is proved by the proclivity to disease—or rather the augmented excitability—remaining in a part that has been once inflamed; and is not the less true, although contrary to the usual laws of habit and the Brunonian doctrine of exhaustion. By this means, a new temperament or idiosyncracy, general or local, may be produced. Inflammation in its duration retains no relation to the operation of its cause, but proceeds much less influenced by the state of the general system, than the system is influenced by it. In a case of mere stimulation or excitement, (as in the condition resulting from violent exercise, ebriety, sun-stroke, &c.) the subtraction of the cause removes the effect; but when once *inflammation* is by any of the same causes lighted up, it will hold its course, in a certain degree at least, however the cause be withdrawn. Inflammation is, therefore, an *independent* process; a fact which is farther proved by its arising in the most debilitated subjects, as after hæmorrhages, and in the last stage of febrile diseases. All authors, from Galen to Darwin, with the exception of Brown, considered inflammation as always a state of encreased action; the *error loci*, the *spasm*, the *obstruction*, &c. &c. of defferent systems, being merely the antecedents or causes of the inflammation, like the *thorn* of Van Helmont, or the *stimulus* of Haller. All practical writers admitted the various *kinds* of inflammation, as modified by the varying state of the general excitement, the character of the part, &c. but still they considered the action of the affected part as increased. Brown alone conceived the idea of asthenic inflammation. It is true that two opposite *general* states of the system (diathesis) cannot co-exist; but the co-existence of a state of *general* debility with a *local* excess of stimulus, and *vice versa*, is very comprehensible. Although local inflammation is, in a certain respect, *independent* of the diathesis, still it is *influenced* by the latter, and *vice versa*. Thus, if the general excitement be morbidly high, this will augment the local inflammation; if the general excitement is moderate, it will be encreased by the local excess of stimulus; and if the general excitement is very low, it will lessen the degree of the local stimulus. It is this last way in which general antiphlogistic treatment relieves local inflammation. In judging of the nature of a disease it is of great importance to distinguish between its primary essential characters, and its ultimate condition, or consequences. Gangrene and sphacelus are assuredly very unlike inflammation in many respects; but there was a time when these very processes were inflammatory and curable, if curable by antiphlogistics. On every account, therefore, both in a pathological and practical point of view, it is of the utmost necessity to watch the beginning of diseases.

> " E duopo abituarci a prevenire con attività i passi ulteriori; ad agir con prontezza, trattandosi di violenti malattie, in que' primi momenti, i soli pur troppo che prestino un filo alla diagnosi, i soli a mio avviso che debbano considerarsi preziosi per l'arte, e per l'umanità." *Dell'Infiammazione, p.* III.

Of the application of these new doctrines to practice, in the Italian school, we are furnished with an interesting exemplification in the third work of Tommasini, named at the head of this article. This, as will be seen by its title, is a brief report of the practice in the clinical wards of the university of Bologna, for the three years ending in 1820, delivered by the author in the form of a lecture to the students. With a transcription of the Professor's list of diseases, and some of his remarks, we shall conclude this article without any farther comment of our own. It must appear to all that the new practice is very superior to the old; but many will be disposed to fear, with ourselves, lest it be carried too far, if it has not been so already. The Italians ought to recollect that their country was once before *inflamed* from a neglect of the maxim—*in medio tutissimus ibis*—and pronounced too by the very god of physic.*

---

* The doses in which medicines are exhibited by the Italian physicians, are so enormously large, as almost to exceed credibility. As a specimen we insert the following list, which, we are assured, was furnished by Dr. Carlo Bellati himself, one of the most distinguished of the physicians of the hospital at Pavia :—

Tartar emetic from half a drachm to three drachms daily.
Extr. of aconite, from two grains to three ounces (he must mean drachms) daily.
Digitalis from two to six grains daily.
Gamboge, from six grains to thirty every three hours.
Cream of tartar, three ounces a day for a month.
Muriate of lime, from a drachm to half an ounce daily.
Extr. of henbane, from two grains to twenty-four every two hours.
Carbonate of soda, a drachm to an ounce daily.
Carbonate of ammonia, half an ounce daily.
Powder of nux vomica, from a grain to one drachm daily.
The same of the extract.

" I can hardly believe," says Rasori, " that the toleration of such large doses of emetic tartar will be attributed to any want of strength in the medicine. The physicians of Genoa know that the tartar emetic of their apothecaries prepared from the glass of antimony, produces vomiting in doses of two or three grains."

In the management of sthenic or inflammatory dropsies, whether anasarca, ascites, or hydrothorax, Rasori gave large doses of the tartrite of antimony, with nitre and diluents, which, joined to abstinence, were pro-

### LIST OF DISEASES.

| | Admitted. | Died. |
|---|---|---|
| Acute Inflammations, including 15 cases of rheumatism and eight of exanthemata | 209 | 21 |
| Chronic inflammations, including 13 cases of dropsy | 38 | 5 |
| Synochal and catarrhal fevers | 35 | — |
| Synochus, nervous, or typhus fevers | 57 | 4 |
| Severe acute diseases from defect of stimulus | 4 | — |
| Simple intermittents, or combined with physconia | 45 | — |
| Hæmorrhages | 17 | 1 |
| Convulsions, including two cases of madness | 18 | 1 |
| Asthmatic Affections | 4 | — |
| Torpor, hemiplegia, and apoplexy | 10 | 1 |
| Decided irritative affections | 10 | — |
| Hydrophobia | 2 | 2 |
| Pellagra | 1 | — |
| *Vizi strumentali* (surgical ?)* | 3 | — |
| Total | 453 | 35 |

"Of the above diseases," the author remarks, "the least severe were the 35 cases of synochal and catarrhal fevers, the 45 intermittents, and the 11 painful, convulsive, or febricular affections, manifestly merely *irritative* in their nature. These last were all cured by the removal of the exciting cause, either by its expulsion or destruc-

---

ductive of the best effects. Some of the patients took daily seven or eight grains of emetic tartar, with an ounce and a half or two ounces of cream of tartar in divided doses. In other cases, thirty grains of jalap with cream of tartar were given in the same way.

Tommasini, however, acknowledges that diseases decidedly sthenic, and requiring the depletive plan, fall sometimes, in the course of the phlogistic process, into a state of temporary but evident "counter-stimulus," in which they will not bear that depletion before tolerated, and subsequently again required This state, he thinks, is often produced by pain, and by some other secret pathological condition, not yet detected. *See Bell, in Dr. Chapman's Journal, No.* 5, *for November,* 1821.

* *Surgical?* We believe that *vizi strumentali,* which the reviewer has queried, does not mean surgical, but *organic* diseases. In a work of Professor Barzellotti, published last year, he has arranged diseases into two classes—those of increased, and those of diminished action. The latter are subdivided into six orders, the fifth of which contains those *vizi strumentali,* the literal translation of which would be "organic derangements," but which he has thus defined: "Derangements of the functions of the most important organs, (namely, of the brain, heart, lungs, and chylopoietic viscera) which have not been produced *primarily* by any disease in the organs themselves, and which are not symptomatic of any of the other diseases before arranged." He then gives a list of the diseases comprehended in the above term, among which we observe asthma, angina pectoris, pertussis, &c. &c. &c. *Ed.*

tion. These cases demonstrated not merely the existence of this class of diseases, but proved with what violence of symptoms a simple affection, not possessing any diathesis, can assume the appearance of a severe malady. The great infrequency of similar affections, however, ought to convince us, that, in most cases, the irritative agency of the first cause, however slight or transient, is soon followed by processes that constitute an affection of diathesis, independent of its cause, and no longer curable by its removal. All the *intermittents* were cured, although in many of these, a chronic inflammation of the liver or spleen, required the long-continued use of antiphlogistic measures. We were equally successful in the catarrhal and synochal affections, although many of these exhibited symptoms which would have rendered the application of depressing measures much less steady in the hands of any practitioner who was not convinced (as I have long been) that all these affections, viz. synocha, synochus, and typhus, are mere varieties and degrees of the same disease; and that it is by the employment of an opposite plan of treatment that the simplest synocha is often converted into the severest typhus." *Prospetto*, pp. 21, 22, 23.

In illustration of this last statement, he adds, in another place—

"It is a fact that, during the period when exciting remedies were so much used in febrile affections, nervous and typhus fevers were not only more frequent, but the mortality from them much greater, being never less than 18 or 20 per cent. The diminution of mortality during the latter years, throughout Italy, since the establishment of the antiphlogistic treatment, has been wonderfully great; and we see in the results of the table, given above, that the mortality in nervous fever, in our clinical wards has been less than eight per cent." P. 25.

He says that acute inflammatory diseases are those most prevalent in Bologna, particularly during the winter. Those admitted into the clinical hospital consisted of a few cases of exanthemata, (which Tommasini considers in all their forms simply as inflammatory affections of the skin and vascular system,) rheumatism, gout, &c. the remaining being affections of the internal viscera. Of these latter, the diseases of the chest were most numerous. Of 115 pneumonic subjects, 14 died; and of the whole 209 cases of acute inflammation, the mortality was 10 per cent. On the subject of blood-letting in these cases the author makes the following remarks:—

"In some cases of uncommon obstinacy, or of relapse, occurring in strong constitutions, the repetition of venesection must keep pace with the pertinacity of the disease. But, in general, I am unwilling to have recourse to an extraordinary number of bleedings, because I am of opinion that there is a term beyond which the continued application of contrastimulants may be necessary, and yet blood-letting not be so; and because I have found so potent a succedaneum in the use of *kermes mineral, nitre, squills, acetate of potass,* and *laurel water.*" P. 27.

In most cases, he says, even the most severe, he rather trusted to the use of remedies *positively sedative*, than to profuse abstraction of blood. He briefly refers to the particulars of many of these cases, wherein blood-letting and other antiphlogistics were freely used, even in the last stages, and with decided benefit even in the most unpromising cases. In one instance of a peripneumony arising during the progress of a most severe nervous fever, he mentions the success obtained from "the continued use of the antiphlogistic regimen, even to the last, and in defiance of the mortal symptoms of physiological debility." In a case of what he calls "acute pneumo-trachitis," he says that, "neither the long continuance of the disease, nor the prostration of strength and emaciation, prevented the continued application of depressing measures, stibiated tartar and kermes in large doses, antiphlogistic beverages, and bleedings patiently repeated;" all of which cured the disease, and prevented the accession of a tracheal phthisis that appeared inevitable. He mentions a third attack of peripneumony in a woman who had been bled *seventeen* times in the former attacks, but who was eventually cured "by the perseverance in the depressing plan, including even venesection." P. 33. In another case he mentions having bled a man *eighteen* times, and with final success. In a case of most severe synochus, he bled *fourteen* times, besides using, at the same time, "various remedies all unequivocally of the class of depressing or contrastimulant." P. 34. We shall conclude with the following case detailed somewhat more at length.

"A woman came into the hospital on the 11th day of a peripneumony, which had been entirely neglected hitherto, and was consequently now very violent. The patient was old, the respiration sonorous, the quantity of expectoration surprizingly great, the pulse small, and the physiological prostration of strength great. In these circumstances, nevertheless—confident in the maxim, (confirmed to me by a thousand cases, and a thousand dissections,) that inflammation is always one and the same, and, where curable, curable only by antiphlogistic means—so far from attempting to excite the depressed powers by stimulants, I employed bleedings proportioned to the patient's condition; and, encouraged by the resulting amendment, repeated them to the fourteenth time, and with complete success."

It may be here necessary to observe that the bleedings mentioned in the above cases were probably what we should call very small, although the author nowhere mentions the quantity of blood taken. Dr. Clark, in his excellent and amusing work on the climate of France and Italy, in speaking of the practice of this very place (Bologna) remarks as follows:—

"Their treatment of pneumonia differs chiefly from ours in taking away smaller quantities of blood at a time, which necessarily requires the evacuation to be more frequently repeated. This does not appear to arise from a fear of taking away too much blood, for they seem liberal enough in the use of the lancet. This practice is not peculiar to Bologna. In going round one of the hospitals of Rome, with the physician, we came to a young plethoric man labouring under inflammation of the lungs. He had been in the hospital twenty-four hours and had been bled three times, twice to seven, and once to six ounces, and had had four blisters applied, one to each arm and one to each thigh. On inquiring what he expected from the numerous blisters, he said they were contrastimulants."—*Medical Notes*, p. 201.

After stating briefly several other cases, no less strikingly illustrative of the new doctrines than the above, he comes to speak of *dropsy ;* and it is truly gratifying to find the pathology and practice in this disease, lately so much the subject of investigation in England, so well understood in Italy.

"Neither is the dropsy curable by any other means, when it is the result of the chronic inflammation of the viscera or glands, or of the superficial phlogosis of the internal secreting surfaces, as is the case in the greater number of instances." P. 43.

"But were there no examples of *asthenic* diseases, namely, diseases produced and maintained by defect of stimulus, or by the *diathesis of contrastimulus?* Such diseases assuredly were very few, as may be seen from the table. This is no fault of ours; but the fault of an erroneous theory that formerly led men to see things otherwise than they were in nature. To prove the rarity of such diseases, it is enough to mention the single fact, that of a hundred dead bodies, from all diseases, 95 will exhibit either actual inflammation, or its traces, or its relics." P. 44.

But we must here conclude this already too extended article—congratulating the medical profession on the progress of a sounder philosophy and practice throughout the world,—congratulating Italy, more especially, on the superiority of her therapeutics over those of many of her continental neighbours—and expressing an earnest hope, that the present fervor of her sons in the cause of contrastimulants and antiphlogistics, may not lead them beyond the bounds of prudence—to be again the victims of their misguided zeal, and to afford once more, amid the ruin wrought by their errors, the occasion for *yet another new doctrine*, to flourish and to fall, like those that have preceded it.

——8, page 747, *for* Lio Scozzes, *read* Lo Scozzes.

